# Quantitative Trait Locus Mapping for Plant Height and Branch Number in CCRI70 Recombinant Inbred Line Population of Upland Cotton (Gossypium hirsutum)

**DOI:** 10.3390/plants13111509

**Published:** 2024-05-30

**Authors:** Gangling Li, Jincan Che, Juwu Gong, Li Duan, Zhen Zhang, Xiao Jiang, Peng Xu, Senmiao Fan, Wankui Gong, Yuzhen Shi, Aiying Liu, Junwen Li, Pengtao Li, Jingtao Pan, Xiaoying Deng, Youlu Yuan, Haihong Shang

**Affiliations:** 1 School of Agricultural Sciences, Zhengzhou University, Zhengzhou 450001, China; ligangling@zzu.edu.cn (G.L.); chejincan@163.com (J.C.); 2 National Key Laboratory of Cotton Bio-Breeding and Integrated Utilization, Institute of Cotton Research, Chinese Academy of Agricultural Sciences, Anyang 455000, China; gongjuwu@caas.cn (J.G.); 13027729012@163.com (L.D.); 15066223517@163.com (X.J.); 15369052883@163.com (P.X.); fsmtmu@163.com (S.F.); gongwankui@caas.cn (W.G.); liuaiying@caas.cn (A.L.); lijunwen@caas.cn (J.L.); lipengtao1056@126.com (P.L.); panjingtao@caas.cn (J.P.); dengxiaoying8@163.com (X.D.); 3 Center for Computational Biology, College of Biological Sciences and Technology, Beijing Forestry University, Beijing 100083, China; 4 National Key Laboratory of Cotton Bio-Breeding and Integrated Utilization, Key Laboratory of Plant Stress Biology, College of Life Science, Henan University, Kaifeng 475001, China

**Keywords:** branch number, candidate gene, high-density genetic map, plant height, quantitative trait locus, upland cotton

## Abstract

Upland cotton accounts for a high percentage (95%) of the world’s cotton production. Plant height (PH) and branch number (BN) are two important agronomic traits that have an impact on improving the level of cotton mechanical harvesting and cotton yield. In this research, a recombinant inbred line (RIL) population with 250 lines developed from the variety CCRI70 was used for constructing a high-density genetic map and identification of quantitative trait locus (QTL). The results showed that the map harbored 8298 single nucleotide polymorphism (SNP) markers, spanning a total distance of 4876.70 centimorgans (cMs). A total of 69 QTLs for PH (9 stable) and 63 for BN (11 stable) were identified and only one for PH was reported in previous studies. The QTLs for PH and BN harbored 495 and 446 genes, respectively. Combining the annotation information, expression patterns and previous studies of these genes, six genes could be considered as potential candidate genes for PH and BN. The results could be helpful for cotton researchers to better understand the genetic mechanism of PH and BN development, as well as provide valuable genetic resources for cotton breeders to manipulate cotton plant architecture to meet future demands.

## 1. Introduction

Cotton is one of the most important cash crops in tropical and sub-tropical regions of the world. Upland cotton (*Gossypium hirsutum* L.) is the most widely cultivated species, which accounts for over 90% of global cotton production, reflecting its wide adaptability and high-yield characteristics [1,2,3]. Plant architecture, defined as the three-dimensional organization of the entire plant, is an important trait that strongly influences plant development and yield [4]. Cotton plant architecture includes the plant height, branch number, height of the node of first fruiting branch and the angle between the stem and fruiting branch, etc. [5]. Appropriate cotton plant architecture can improve the number of cotton bolls and their opening at right time and allows for increased planting density, thus increasing the level of cotton mechanical harvesting and yields [6,7]. Plant height, which shows dynamic development and heterosis, is a major trait affecting the plant biomass yield, harvest index and economic yield [8]. Branch number is an important agronomic trait of cotton crops and largely influences the morphological structure, photosynthetic capacity, planting density and yield of upland cotton [9]. Plant architecture traits including PH and BN are mainly quantitative traits and are greatly influenced by the environment [10]. Therefore, improving PH and BN with traditional breeding methods may have a low efficiency and take more time in the field. On the contrary, marker-assisted selection (MAS) may play an important role in improving PH and BN more efficiently. Hence, discovering more QTLs of PH and BN will be useful for cotton plant architecture breeding.

Previous studies identified numerous QTLs for PH and BN using different kinds of markers such as simple sequence repeat (SSR), amplified fragment length polymorphisms (AFLPs) and random amplified polymorphic DNA (RAPD) [8,10,11,12,13,14,15]. Compared to these traditional highly labor-intensive and time-consuming markers, single nucleotide polymorphisms (SNPs) exhibit the most plentiful and stable genetic variations in the genome [16]. Previous studies using SNP maps primarily focused on fiber quality and yield traits [17,18,19,20,21,22,23], while only a few studies use SNP maps for PH and BN [11,15,24,25]. In Ma’s research, four stable QTLs for PH were obtained using an SNP-based high-density genetic linkage map [11]. *GhPIN3* is a candidate gene located in the stable QTL *qPH-Dt1-1* region, which encodes an auxin efflux vector protein. Virus-induced gene silencing (VIGS) experiments have shown that *GhPIN3* has a significant impact on plant height [11]. Therefore, using an SNP-based high-density genetic linkage map for PH and BN QTL mapping is an effective method and necessary for improving the accuracy of QTL localization, identifying novel stable QTLs and exploring the genetic basis of the cotton plant height and branch number.

In this study, a recombinant inbred line (RIL) population of 250 lines was used to construct a high-density genetic map with 8298 SNP markers developed with a CottonSNP80K array [26]. The QTLs for PH and BN were identified using the phenotype data from nine and eight environments, respectively. Finally, 9 stable QTLs were identified for PH and 11 stable QTLs for BN. It is worth mentioning that only one stable QTL for PH had been reported in the previous studies. The genes located on the confidence intervals of stable QTLs could be considered as potential candidate genes. Based on the annotation information of gene ontology (GO) and Kyoto Encyclopedia of Genes and Genomes (KEGG) database and the expression pattern data in Zhang’s research [2], the candidate genes were finally identified. Our findings provide novel genetic information on the regulation of PH and BN development, and lay a promising foundation for ideal cotton plant architecture breeding to meet the future demand for mechanical harvesting technology.

## 2. Materials and Method

### 2.1. Plant Materials

CCRI70 (Zhongmiansuo70), an F_1_ hybrid developed from a cross using transgenic insect-resistant cotton line sGK156 as the female parent and high-quality line 901-001 as the male parent, was certified as the national variety in 2008 [27]. sGK156 was developed by the Semi-arid Agriculture Engineering and Technology Research Center of China and Institute of Cotton Research, CAAS. This variety is high-yielding cotton. The other parent, 901-001, produces a high-quality fiber due to introgression from *Gossypium barbadense* to *Gossypium hirsutum* [27]. CCRI70 has an excellent fiber quality with an average fiber length of 32.5 mm, fiber strength of 33.5 cN/Tex and a micronaire of 4.3. The process of developing the CCRI70 recombinant inbred lines population has been described in previous work [28].

In the year 2015, the RIL population was planted in Anyang (2015AY) of the Henan Province, Linqing (2015LQ) of the Shandong Province and Alaer (2015ALE) of the Xinjiang Autonomous Region of China. In the year 2016, it was planted in Anyang of the Henan Province (2016AY), Linqing (2016LQ) of the Shandong Province, Changde (2016CD) of the Hunan Province, Alaer (2016ALE), Shihezi (2016SHZ) and Kuerle (2016KEL) of the the Xinjiang Autonomous Region of China. The RILs and their parents were planted in two replications at the cited experimental sites following a randomized incomplete block design. The plant material was grown in two-row plots where each row was 3 m in length and a 0.8 m distance was kept between the rows in Anyang, while single-row plots, with 0.8 m row distances and 5 m row lengths, were used in Linqing. Four (narrow) row plots of 3 m in length and 0.1 m or 0.66 m (alternating) were maintained between the rows in Kuerle. Three-row plots with a 3.5 m length of each row and 0.76 m between rows were kept in Shihezi. Finally, two-row plots were kept in Chengde with a 3 m length of each row and 1 m distance maintained between the rows. For PH, phenotypic data for all nine environments was collected. For BN, eight environmental data are collected (except for 2015LQ).

### 2.2. Collection and Statistical Analysis of Phenotypic Data

The phenotype data of plant height (PH) and branch number (BN) were collected in September, and 10 plants were selected in each plot. PH was measured from the cotyledonary node to the apex of stem. BN was counted for all the branches of the randomly selected plant. One-way ANOVA and Microsoft Excel were used to test the significance of the difference in PH and BN between two parents. SPSS20.0 was used for the analysis of the average, standard deviation, skewness, kurtosis and correlation of PH and BN in the population.

### 2.3. High-Density Map Construction and QTL Identification for PH and BN

The linkage genetic map is based on the TM-1 reference genome using HighMap software [29,30]. Detailed information on ordering SNP markers and correcting genotypic errors for these chromosomes can be found in Liu’s research [30]. The algorithm SMOOTH was used to correct the error based on parental contribution, and the missing genotypes were imputed by a k-nearest neighbor algorithm [31,32]. The skewed markers were added to the linkage map by a multipoint method of maximum likelihood. The map distances were estimated by the mapping function Kosambi [33].

The software WinQTLCart 2.5 was used to identify QTLs of PH and BN with the composite interval mapping (CIM) method [34,35]. The logarithm of odds (LOD) value for declaring significant QTLs across environments was calculated by a permutation test with the mapping step of 1.0 cMs, five control markers and a significance level of *p* < 0.05, n = 1000. The rules of naming QTL are as follows: the name of QTL starts with “q”, followed by abbreviation of trait, chromosome number and QTL sequence number [36].

The stable QTLs were compared with the CottonQTLdb database (http://www.cottonqtldb.org, accessed on 15 May 2023) to determine whether they were novel. The stable QTLs in this study that shared the same or overlapping confidence intervals in the previous studies were considered as common QTLs.

### 2.4. Gene Identification and Annotation

All the potential candidate genes were predicted by comparing the corresponding homologous genes in *Arabidopsis* (TAIR10) and also annotated with the GO (http://archive.geneontology.org/latest-lite/, accessed on 21 August 2023 and ftp://ftp.ncbi.nlm.nih.gov/gene/DATA/, accessed on 21 August 2023) and KEGG database. The BLASTX software was used to compare the sequences of the candidate genes with the sequences in the database. The results with e values less than e^−10^ were considered to be significant. The KEGG annotation information was obtained from the KOBAS 3.0 software [37].

### 2.5. In-Silico RNA-Seq Data Analysis

RNA-seq data for different tissues of upland cotton were downloaded from the Sequence Read Archive (SRA) of the National Center for Biotechnology Information (NCBI) (https://www.ncbi.nlm.nih.gov/, accessed on 30 August 2023, accession codes. SRA: PRJNA248163) [2]. The software HISAT2 v2.1.0 [38] was used to compare the RNA-seq reads in Zhang’s research [2] into the genome of upland cotton. The fragments per kilobase of exon per million reads (FPKM) values of genes were quantized by StringTie v1.3.5. Genes with an FPKM value greater than 10 in at least one tissue could be considered as expression genes [15].

## 3. Results

### 3.1. Statistical Analysis of PH and BN

**Figure 1:**
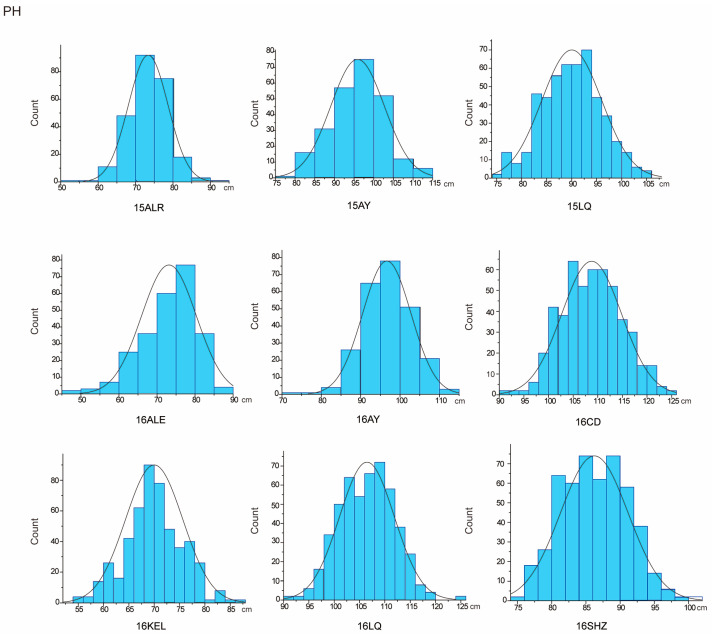
PH approximate normal distribution analysis.

**Figure 2:**
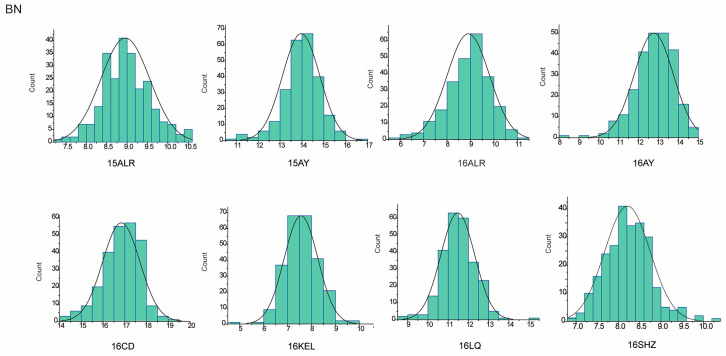
BN approximate normal distribution analysis.

The descriptive statistics of PH for nine environments and BN for eight environments in parents and the RIL population were summarized in Table 1. For the parent sGK156, the average values of PH in nine environments and BN in eight environments were 86.39 cm and 11.39, respectively, and for the parent 901-001, they were 92.91 cm and 11.34, respectively. For the RIL, the average value of PH in nine environments is 88.95 cm. BN only included eight environmental data, the average value of BN in eight environments is 11.1. In addition, both traits exhibited approximately normal distributions, with the absolute skewness values no more than 1 (Table 1) and showed excessive bias in the performance of their parents (Figure 1 and Figure 2). Through the analysis of the correlation between the various environments of PH and BN, it was found that for PH, except for 15LQ and 15ALE, 15AY and 16ALE, the environments all exhibited extremely significant or significant correlation. For BN, except for 16CD and 15ALE, 16ALE and 15AY, 16CD and 16ALE, 16SHZ and 16ALE, 16LQ and 16CD, 16SHZ and 16CD, the other environments all exhibited extremely significant or significant correlations (Table S1).

### 3.2. QTL Identification

**Figure 3:**
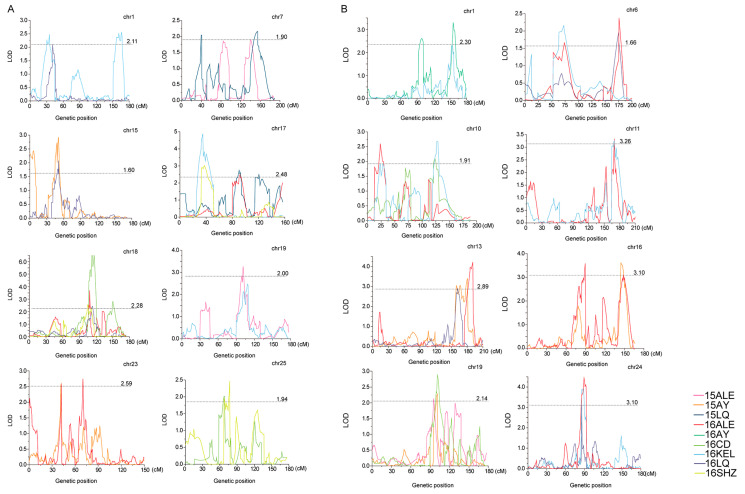
Positions of stable QTLs in plant height and branch number. (**A**) Positions of stable QTLs in plant height. (**B**) Positions of stable QTLs in branch number.

The QTLs were identified with a high-density SNP-based genetic map constructed by Zou [28], which contained 8298 SNP markers spanning a total distance of 4876.70 cMs over 26 chromosomes with an average marker interval of 1.09 cMs (Table S2 and Figure S1). For PH, except for chromosomes 3, 13 and 14, there are 69 QTLs on the other 23 chromosomes (Table 2 and Figure 3A). The QTLs identified in at least two environments with the same or overlapping confidence intervals for the same trait can be considered as stable QTLs [36]. Finally, nine stable QTLs for PH were identified. The QTL *qPH-chr18-2* was detected in four environments, located on CIs of 108.0–116.3 cMs of chr18, and explained 3.84–12.85% of observed phenotypic variation (PV) with negative additive effects. The QTL *qPH-chr17-2* was detected in three environments, located on the CI of 31.9–46.6 cMs of chr17, and explained 5.26–8.69% of the observed PV with negative additive effects. The QTLs *qPH-chr1-2*, *qPH-chr7-4*, *qPH-chr15-1*, *qPH-chr17-2*, *qPH-chr17-3*, *qPH-chr19-3*, *qPH-chr23-2* and *qPH-chr25-1* were detected in two environments, located on CIs of 31.40–45.80 cMs of chr1, 127.7–161.7 cMs of chr7, 48.2–53.6 cMs of chr15, 31.9–46.6 cMs of chr17, 84.3–97.2 cMs of chr17, 93.5–111.1 cMs of chr19, 40.6–47.5 cMs of chr23 and 58.2–70.3 cMs of chr25 and explained 3.76–4.37%, 3.24–3.70%, 2.77–5.08%, 5.26–8.69%, 4.18–4.78%, 3.52–4.23%, 4.16–4.59% and 3.35–3.61% of the observed PV, respectively, all with negative additive effects.

For BN, except for chromosomes 2 and 3, there are 63 QTLs on the remaining 24 chromosomes (Table 2 and Figure 3B). Eleven stable QTLs were finally identified. The QTLs *qBN-chr19-3* and *qBN-chr24-1* were detected in three environments, located on CIs of 92.3–104.9 cMs of chr19 and 85.1–93.0 cMs of chr24, and explained 3.61–5.03% and 5.47–7.75% of the observed phenotypic variation (PV), respectively, both with negative additive effects. The QTLs *qBN-chr1-5*, *qBN-chr6-1*, *qBN-chr6-2*, *qBN-chr10-1*, *qBN-chr10-2*, *qBN-chr11-2*, *qBN-chr13-3*, *qBN-chr13-4* and *qBN-chr16-5* were detected in two environments, located on the CIs of 151.9–160.3 cMs of chr1, 52.5–85.3 cMs of chr6, 166.3–181.2 cMs of chr6, 19.3–30.8 cMs of chr10, 120.8–131.3 cMs of chr10, 164.5–171.8 cMs of chr11, 157.5–167.4 cMs of chr13, 176.2–192.4 cMs of chr13 and 143.8–154.7 cMs of chr16 and explained 4.2–5.64%, 2.9–3.74%, 3.81–4.14%, 3.22–4.37%, 4.18–4.65%, 5.49–5.50%, 5.00–5.19%, 6.18–7.39% and 4.88–5.99% of the observed PV, respectively. The negative additive effects were for *qBN-chr6-1*, *qBN-chr6-2*, *qBN-chr10-2*, *qBN-chr13-3*, *qBN-chr13-4* and *qBN-chr16-5*, and the positive additive effects were for *qBN-chr1-5*, *qBN-chr10-1* and *qBN-chr11-2*.

### 3.3. Gene Identification, Annotation and Expression Pattern Analysis

**Figure 4:**
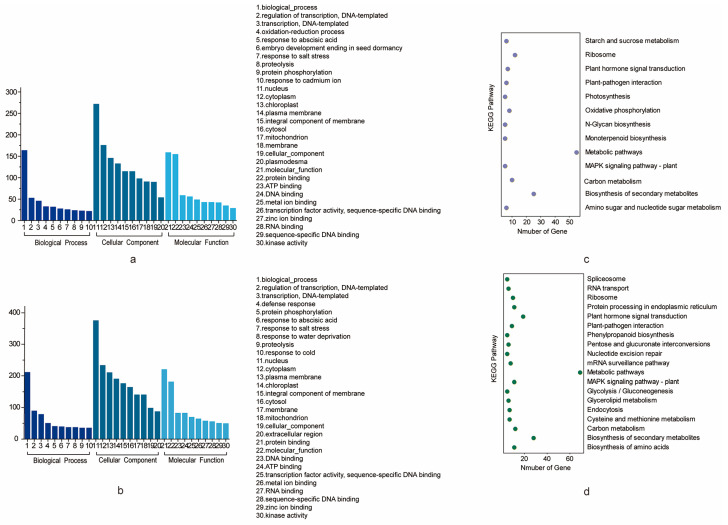
GO and KEGG analysis of the potential candidate genes. (**a**) GO analysis of the potential candidate genes for PH. (**b**) GO analysis of the potential candidate genes for BN shows terms containing at least 10 genes. (**c**) KEGG analysis of the potential candidate genes for PH. (**d**) KEGG analysis of the potential candidate genes for BN.

All the genes that are located in the confidence interval of the stable QTLs for PH and BN could be considered as potential candidate genes [28]. A total of 941 genes (495 for PH and 446 for BN) located on the CI of stable QTLs were used for further analysis. The physical location intervals corresponding to some genetic confidence intervals were too long, so we reasonably divided them into two relatively short intervals. For PH, *qPH-chr15-1* covered a maximum of 101 genes, while *qPH-chr17-2* covered a minimum of 15 genes (Table 3). For BN, *qBN-chr6-1* covered a maximum of 83 genes, while *qBN-chr10-1* covered a minimum of one gene (Table 3). The functions of the PH and BN genes were achieved by identifying the corresponding genes in *Arabidopsis* (Table S3). Genes are also annotated with GO and KEGG (Figure 4). For GO annotation, the GO terms “biological process” in the category “biological process”, “nucleus” in the category “cellular component” and “protein binding” in the category “molecular function” harbored the most genes for both traits. For PH, these three GO terms harbored 130, 215 and 127 genes. For BN, these three GO terms harbored 103, 201 and 106 genes, respectively (Table S4). For KEGG annotation, the KEGG pathways “Metabolic pathways”, “Biosynthesis of secondary metabolites”, “Carbon metabolism” and “Ribosome” harbored the most genes of PH. These four pathways harbored 39, 20, 9 and 9 genes, respectively. The KEGG pathway “Metabolic pathways”, “Biosynthesis of secondary metabolites”, “MAPK signaling pathway-plant” and “Plant-pathogen interaction” harbored the most genes of BN. These three pathways harbored 34, 13 and 8 genes, respectively (Table S5).

Fifty-nine genes for PH were expressed in at least one of the three tissues (root, stem and leaf) according to the RNA-seq data [2]. Amongst them, 23 genes were expressed in all three tissues, while 27 genes were expressed in stems and leaves and 24 genes were expressed in roots and stems; 28 genes were expressed in roots and leaves, 5 genes were only expressed in roots and 14 genes were only expressed in stems, and 6 genes were only expressed in the leaves (Table S6). For BN, 67 genes were expressed in at least one of the three tissues (root, stem and leaf). Among them, 20 genes were expressed in all three tissues, 27 genes were expressed in stems and leaves and 25 genes were expressed in roots and stems; 27 genes were expressed in roots and leaves, 8 genes were only expressed in roots and 15 genes were only expressed in stems, and 5 genes were only expressed in the leaves (Table S6).

## 4. Discussion

### 4.1. Phenotypic Evaluation

Both PH and BN are quantitative traits and are easily affected by the environment. The phenotypic data of PH and BN exhibit a normal distribution, aligning with the characteristics of quantitative traits and can be used for further QTL mapping. The correlation analysis of each environment concluded that significant correlations exist between most of the environments, while there are still relatively poor correlations between individual environments. One of the possible reasons could be the significant differences in climate and soil types among the cotton areas in the Yellow River Basin, the Yangtze River Basin and the northwest inland cotton areas, and PH and BN are greatly affected by environmental factors. The development of PH and BN involves interactions between environmental and genetic factors, in which many genes often interact with one another and with environmental factors and in no additive pathways together [39]. Therefore, it is necessary to carry out multi-environment experiments to improve the accuracy of QTL positioning.

### 4.2. Genetic Map Construction

In past research there are many types of molecular markers (SSR, RFLP and SNP), which are used for the construction of genetic maps. But there were some pitfalls: firstly, the physical locations in the genetic maps constructed using SSR and RFLP molecular markers that appeared before the reference genome were inaccurate. Secondly, its map coverage was low and always had large gaps. Therefore, using these maps to identify QTLs may result in larger confidence intervals, ultimately leading to more candidate genes being obtained. However, SNP markers, which were developed from next-generation sequencing technology and genotyped with the reference genome, provided a high-density genetic map with unprecedented accuracy. In this study, a high-density genetic map containing 8298 SNP markers, with a total genetic distance of 4876.70 cMs and an average marker interval of 1.19 cMs, was used to identify QTLs. The high-density SNP markers almost covered the whole genome of upland cotton. It serves as a valuable tool for QTL identification, candidate gene analysis and molecular breeding of upland cotton.

### 4.3. Congruence with Previously Reported QTLs

We have noticed that the phenotypic variation between the two parents is minor. At the beginning, we doubted whether the CCRI70 population was suitable for QTL mapping. After thorough mining of the literature, we found that researchers have identified eight grain size genes in rice using populations constructed by minor-phenotypic-difference parents [40]. Finally, we identified 9 stable QTLs for PH and 11 stable QTLs for BN using the CCRI70 population. Then these stable QTLs were compared with the QTLs in cottonQTLdb to show whether the stable QTLs in our study are novel or have been identified previously [41,42,43,44,45,46,47,48]. All markers in the database are SSR markers and restriction fragment length polymorphism (RFLP) markers. Because no universal markers could be found between the SNP maps and the SSR and RFLP maps in the database, meta-analysis cannot be used. QTLs in the database covering a fully or partially overlapped physical CIs with our results are common QTLs. For PH, the QTLs in the database are distributed on 23 chromosomes, except for chromosomes 4, 12 and 18. In our study, we identified a stable QTL on chromosome 18. For BN, the QTLs in the database are distributed on 14 chromosomes, except for chromosomes 3, 4, 5, 6, 7, 13, 15, 16, 18, 19, 20 and 21. In our study, we also identified stable QTLs on chromosomes 6, 13, 16 and 19. By comparing the physical location between the QTL in our research and the QTL in the database, one stable QTL for PH (*qPH-chr25-1*) can be regarded as a common QTL. The other nine stable QTLs for PH and eleven stable QTLs for BN were newly identified. But most QTLs have a minor effect on the observed phenotypic variation (Table 2). These results were similar to Chandnani’s research, in which over 90% QTLs identified using reciprocal interspecific introgression population had small effects (%PV < 10) [25]. This may be because the two parents shared the same major QTLs, making the minor QTLs easier to identify. The other reason for less overlapping with the position of the previous study may be due to the use of the latest reference genome in our study and the smaller final positioning interval. Using the SNP map, we have identified four important QTLs, including *qPH-chr17-2*, *qPH-chr18-2*, *qBN-chr13-4* and *qBN-chr24-1*, which can explain more than 7% of the observed PV in multiple environments. These results provide useful information for further cotton plant architecture breeding by MAS.

### 4.4. Identification of Candidate Genes

For PH, the gene *GH_D03G0586* located in *qPH-chr17-3* encodes a cytochrome P450 protein involved in brassinosteroid biosynthesis pathway. *GH_D03G0586* is expressed in root and leaf according to the expression pattern analysis and verified by the Cotton Omics Database (http://cotton.zju.edu.cn/10.rnasearch.html, accessed on 26 April 2024) (Table S6 and Figure S2) [49]. Mutations in this kind of gene may prevent brassinosteroid biosynthesis, resulting in a dwarf plant [50,51]. *GH_A01G1023* was located in *qPH-chr1-2* and annotated as catalase 2 (CAT2) in *Arabidopsis thaliana*. *GH_A01G1023* is highly expressed in the root, stem and leaf (Table S6 and Figure S3). The previous work indicated that CAT2 was the major enzyme involved in detoxifying ROS in the photosynthetic tissues. It can reduce the accumulation of ROS, thereby accelerating the growth of plants [52,53,54]. Two candidate genes, namely *GH_A01G1055* and *GH_D03G1142*, were enriched into the carbon metabolism and glycolysis/gluconeogenesis pathway and are located in *qPH-chr1-2* and *qPH-chr17-2*, respectively. *GH_A01G1055* and *GH_D03G1142* are both expressed in the root, stem and leaf (Table S6 and Figures S4 and S5). Enolase is the rate-limiting enzyme of the glycolysis/gluconeogenesis pathways, and it also participates in the functional regulation of many metabolic pathways [55,56].

For BN, the gene *GH_A13G0765* located in *qBN-chr13-3* belongs to the WRKY family, which was identified as an important inhibitor of internode elongation in cotton [57,58]. *GH_A13G0765* is only expressed in the stem (Table S6 and Figure S6), indicating its important role in branching formation. AtWRKY71 plays an important role in shoot branches in *Arabidopsis thaliana* by regulating *RAX* genes and auxin signaling [59]. It is possible that *GH_A13G0765* may have a similar effect on shoot branching in cotton. Five candidate genes (*GH_D05G0467*, *GH_D05G0561*, *GH_D05G0383*, *GH_D05G0468* and *GH_D07G0421*) were enriched into plant hormone signal transduction pathway. *GH_D07G0421* located in *qBN-chr16-5* was enriched into MAPK signaling pathway. *GH_D07G0421* is also only expressed in the stem (Table S6 and Figure S7). The available evidence suggests that MAPK cascades are involved in ABA signaling, while studies in cotton indicate that ABA moves into fruiting branches and growing points and inhibits growth [60,61].

All in all, these six genes *GH_D03G0586*, *GH_A01G1023*, *GH_A01G1055*, *GH_D03G1142*, *GH_A13G0765* and *GH_D07G0421* can be considered as candidate genes. Our results have important implications for further exploring the genetic mechanism of cotton plant type determination and for ideal cotton plant architecture breeding by pyramiding such stable QTLs.

## 5. Conclusions

To explore the genetic mechanisms under the development of PH and BN, QTL mapping was carried out using the CCRI70 RIL population with 250 lines. A total of 69 QTLs for PH (9 stable) and 63 for BN (11 stable) were identified using a high-density SNP-based genetic map. By comparing the physical location of the stable QTLs identified in this study with the CottonQTLdb database, we found that only one stable QTL for PH was reported in the previous studies. The genes located on the CI of stable QTL were analyzed through annotation information, expression patterns and previous studies. Six candidate genes (*GH_D03G0586*, *GH_A01G1023*, *GH_A01G1055*, *GH_D03G1142*, *GH_A13G0765* and *GH_D07G0421*) were finally identified. Our results are of great significance for further understanding the genetic mechanism under cotton plant architecture, and lay a promising foundation for the use of MAS for ideal cotton plant architecture breeding.

## Figures and Tables

**Table 1:** The results of the statistical analysis of PH and BN in the parents and population.

		Parents			Population							
Trait	Environment	sGK156	901-001	Range	Average	Standard Error	Standard Deviation	Variance	Kurtosis	Skewness	Minimum	Maximum
	15ALE	77.20	76.60	0.60	73.39	0.34	5.30	28.10	0.83	−0.01	54.50	93.85
	15AY	95.39	94.97	0.42	95.89	0.43	6.81	46.31	−0.07	−0.07	76.26	114.30
	15LQ	96.10	113.90	17.80	90.02	0.38	6.00	35.98	−0.23	−0.02	75.20	105.70
PH	16ALE	72.30	78.40	6.10	73.14	0.45	7.19	51.70	0.63	−0.74	45.90	88.15
	16AY	88.00	94.60	6.60	96.63	0.39	6.15	37.78	0.72	−0.27	72.69	111.88
	16CD	107.40	116.90	9.50	108.76	0.38	6.07	36.80	−0.34	0.04	90.88	124.20
	16KEL	65.75	72.25	6.50	70.00	0.36	5.63	31.75	0.20	0.03	54.30	86.20
	16LQ	95.80	107.80	12.00	106.47	0.34	5.39	29.08	−0.10	0.06	90.95	124.80
	16SHZ	79.55	80.75	1.20	86.22	0.31	4.94	24.36	−0.42	0.12	74.75	101.70
	15ALE	9.55	9.05	0.50	8.96	0.04	0.60	0.36	0.20	0.18	7.25	10.50
	15AY	14.35	12.77	1.58	13.96	0.05	0.85	0.73	1.89	−0.69	10.57	16.63
	16ALE	9.30	9.85	0.55	8.91	0.06	0.91	0.82	0.42	−0.46	5.90	11.30
BN	16AY	12.45	12.00	0.45	12.76	0.06	0.98	0.96	1.47	−0.71	8.25	14.85
	16CD	17.40	18.45	1.05	16.84	0.05	0.86	0.73	0.37	−0.39	14.30	19.45
	16KEL	8.35	8.35	0.00	7.56	0.04	0.70	0.50	1.12	−0.05	4.56	9.95
	16LQ	11.65	12.30	0.65	11.47	0.05	0.84	0.70	1.72	0.11	8.80	15.20
	16SHZ	8.05	7.95	0.10	8.21	0.03	0.54	0.29	0.76	0.43	6.95	10.25

**Table 2:** Stable QTLs for PH and BN.

Name	Chromosome	Environment	Position	LOD	Additive	R2 (%)	Left CI	Right CI
*qPH-chr1-2*	chr1	16KEL	34.71	2.49	−1.18	4.37	31.40	39.40
		16LQ	40.71	2.11	−2.16	3.76	36.20	45.80
*qPH-chr7-4*	chr7	15ALE	139.71	1.90	−1.49	3.24	127.70	149.40
		15LQ	154.71	2.16	−1.35	3.70	140.20	161.70
*qPH-chr15-1*	chr15	15AY	50.61	2.93	−1.56	5.08	48.20	51.90
		16LQ	50.71	1.60	−1.75	2.77	50.60	53.60
*qPH-chr17-2*	chr17	16KEL	34.91	4.89	−2.08	8.69	31.90	35.50
		16SHZ	37.11	3.02	−1.17	5.26	32.40	46.60
*qPH-chr17-3*	chr17	15LQ	90.31	2.74	−1.88	4.78	84.30	93.60
		16ALE	92.11	2.48	−1.68	4.18	89.30	97.20
*qPH-chr18-2*	chr18	16ALE	108.21	3.70	−3.52	6.37	108.00	114.10
		16SHZ	111.41	2.28	−1.27	3.84	107.00	113.90
		16CD	113.51	6.68	−3.15	12.85	111.90	114.20
		16LQ	113.51	2.43	−1.41	4.41	108.10	116.30
*qPH-chr19-3*	chr19	15ALE	108.01	2.00	−1.66	3.52	104.80	111.10
		16KEL	108.51	2.47	−1.29	4.23	105.80	110.00
*qPH-chr23-2*	chr23	15AY	41.91	2.60	−2.17	4.59	40.60	47.50
		16ALE	41.91	2.59	−2.17	4.16	41.30	43.10
*qPH-chr25-1*	chr25	16CD	67.41	2.02	−1.73	3.61	58.20	69.10
		16SHZ	67.41	1.94	−1.38	3.35	66.70	70.30
*qBN-chr1-5*	chr1	16KRL	154.01	2.3	0.20	4.20	151.90	157.10
		16AY	155.81	3.3	0.22	5.64	153.80	160.30
*qBN-chr6-1*	chr6	16KRL	72.61	2.16	−0.18	3.74	52.50	83.00
		16ALE	73.41	1.66	−0.19	2.90	68.60	85.30
*qBN-chr6-2*	chr6	15LQ	175.31	1.95	−0.36	3.81	166.30	180.40
		16ALE	175.91	2.38	−0.43	4.14	175.30	181.20
*qBN-chr10-1*	chr10	16ALE	23.31	2.6	0.24	4.37	19.30	30.80
		16KEL	23.31	1.91	0.22	3.22	22.80	25.00
*qBN-chr10-2*	chr10	16CD	123.91	2.09	−0.15	4.18	120.80	130.80
		16KEL	127.41	2.67	−0.20	4.65	120.90	131.30
*qBN-chr11-2*	chr11	16ALE	169.71	3.33	0.35	5.49	168.60	170.90
		16KEL	169.71	3.26	0.30	5.50	164.50	171.80
*qBN-chr13-3*	chr13	15AY	159.91	3.04	−0.25	5.19	157.50	163.20
		16LQ	161.51	2.89	−0.19	5.00	157.90	167.40
*qBN-chr13-4*	chr13	15AY	180.81	3.39	−0.27	6.18	176.20	183.70
		16ALE	190.81	4.23	−0.33	7.39	183.20	192.40
*qBN-chr16-5*	chr16	15ALE	144.61	3.61	−0.20	5.99	143.80	149.80
		15AY	148.51	3.1	−0.20	4.88	144.00	154.70
*qBN-chr19-3*	chr19	15AY	95.31	2.14	−0.20	3.61	92.30	98.70
		15ALE	101.01	2.29	−0.17	4.09	95.30	104.60
		16CD	101.01	2.9	−0.19	5.03	97.60	104.90
*qBN-chr24-1*	chr24	16ALE	85.81	3.1	−0.33	5.47	85.10	86.10
		16KEL	88.71	3.92	−0.22	6.86	85.50	90.00
		16LQ	89.41	4.45	−0.22	7.75	85.40	93.00

**Table 3:** Genes located on the CI of stable QTLs.

Trait	QTL Name	Genetic Interval (cMs)	Range	Marker Interval	Physical Interval (MB)	Range	Number of Genes
	*qPH-chr1-2*	31.4–45.8	14.40	Marker86-Marker28	19.25–21.52	2.27	73
	*qPH-chr7-4*	127.7–161.7	34	Marker1493-Marker1494	7.87–8.01	0.14	8
				Marker1491-Marker1479	29.04–32.89	3.85	78
PH	*qPH-chr15-1*	48.2–53.6	5.4	Marker5089-Marker5086	17.59–19.87	2.28	101
	*qPH-chr17-2*	31.9–46.6	14.7	Marker5756-Marker5746	39.49–40.07	0.58	15
	*qPH-chr17-3*	84.3–97.2	12.9	Marker5905-Marker5886	8.78–12.71	3.93	85
	*qPH-chr18-2*	107–116.3	9.3	Marker6098-Marker6094	5.66–6.49	0.83	42
	*qPH-chr19-3*	104.8–111.1	6.3	Marker6294-Marker6283	29.99–30.54	0.55	22
	*qPH-chr23-2*	40.6–47.5	6.9	Marker6865-Marker6860	42.47–43.01	0.54	38
	*qPH-chr25-1*	58.2–70.63	12.43	Marker8085-Marker8077	5.01–5.65	0.64	33
	*qBN-chr1-5*	151.9–160.3	8.4	Marker270-Marker268	2.19–2.39	0.2	18
	*qBN-chr6-1*	52.5–85.3	33	Marker1171-Marker1168	100.04–100.81	0.77	83
	*qBN-chr6-2*	166.3–181.2	14.9	Marker1192-Marker1190	11.17–13.49	2.32	32
	*qBN-chr10-1*	19.3–30.8	11.5	Marker2804-Marker2797	94.53–94.60	0.07	1
BN	*qBN-chr10-2*	120.8–131.3	10.5	Marker2886-Marker2860	69.92–71.11	1.19	27
	*qBN-chr11-2*	164.5–171.8	7.3	Marker3124-Marker3117	8.16–8.71	0.55	42
	*qBN-chr13-3*	157.5–167.4	9.9	Marker4162-Marker4152	14.48–17.00	2.52	74
	*qBN-chr13-4*	176.2–192.4	16.2	Marker4206-Marker4199	6.98–11.10	4.12	46
	*qBN-chr16-5*	143.8–154.7	10.9	Marker5701-Marker5686	4.02–4.87	0.85	72
	*qBN-chr19-3*	92.3–104.9	12.6	Marker6280-Marker6275	49.3–50.32	1.02	20
	*qBN-chr24-1*	85.1–93	7.9	Marker7307-Marker7293	46.52–47.50	0.98	31

## Data Availability

The datasets generated during and/or analyzed during the current study are available from the corresponding author upon reasonable request. The data are not publicly available due to privacy.

## Supplementary Materials

The following supporting information can be downloaded at: https://www.mdpi.com/article/10.3390/plants13111509/s1, Table S1-1. Correlation analysis between PH in nine environments and Table S1-2. Correlation analysis between BN in eight environments. Table S2. Detailed information on the linkage map for the CCRI70 population. Table S3. The function of the potential candidate genes in *Arabidopsis*. Table S4-1. The gene list of each GO terms for PH. Table S4-2. The gene list of each GO terms for BN. Table S5-1. Gene number in each KEGG pathway for PH. Table S5-2. Gene number in each KEGG pathway for BN. Table S6-1. The potential candidate genes for PH expressed in three tissues (roots, stems and leaves). Table S6-2. The potential candidate genes for BN expressed in three tissues (roots, stems and leaves). Figure S1. The distribution of SNP markers in the genetic map. Figure S2. The expression pattern of *GH_D03G0586* in the Cotton Omics Database. Figure S3. The expression pattern of *GH_A01G1023* in the Cotton Omics Database. Figure S4. The expression pattern of *GH_A01G1055* in the Cotton Omics Database. Figure S5. The expression pattern of *GH_D03G1142* in the Cotton Omics Database. Figure S6. The expression pattern of *GH_A13G0765* in the Cotton Omics Database. Figure S7. The expression pattern of *GH_D07G0421* in the Cotton Omics Database.
