# Supplementary figures and images for: Quantitative Trait Locus Mapping for Plant Height and Branch Number in CCRI70 Recombinant Inbred Line Population of Upland Cotton (Gossypium hirsutum)
Source: Plants (Basel). 2024 May 30;13(11):1509. doi: 10.3390/plants13111509 (PMC11174691; doi:10.3390/plants13111509)

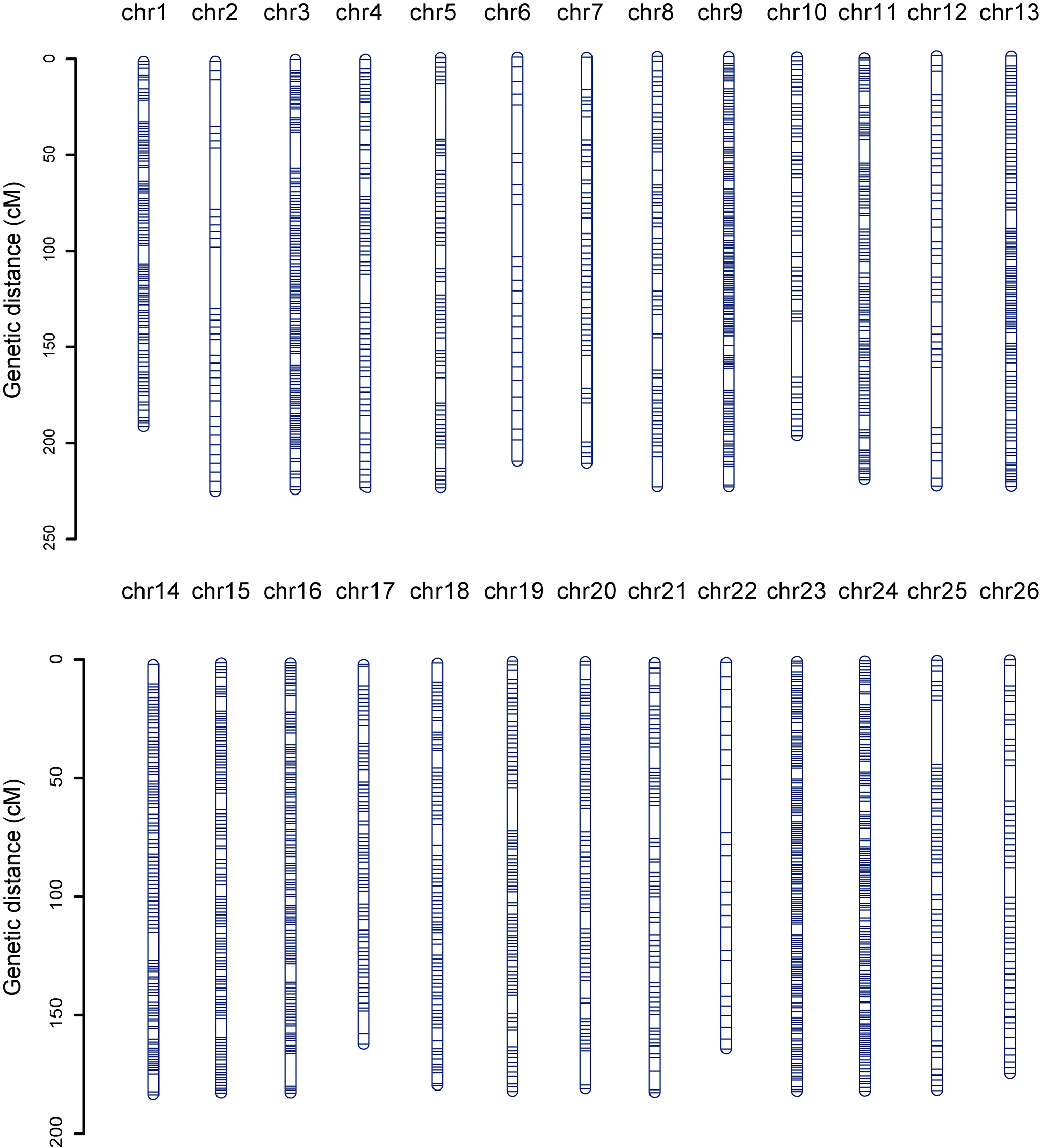

Supplement: Supplementary file 1 [file plants-13-01509-s001.zip › figure.S1 The distribution of SNP markers in the genetic map..tif]

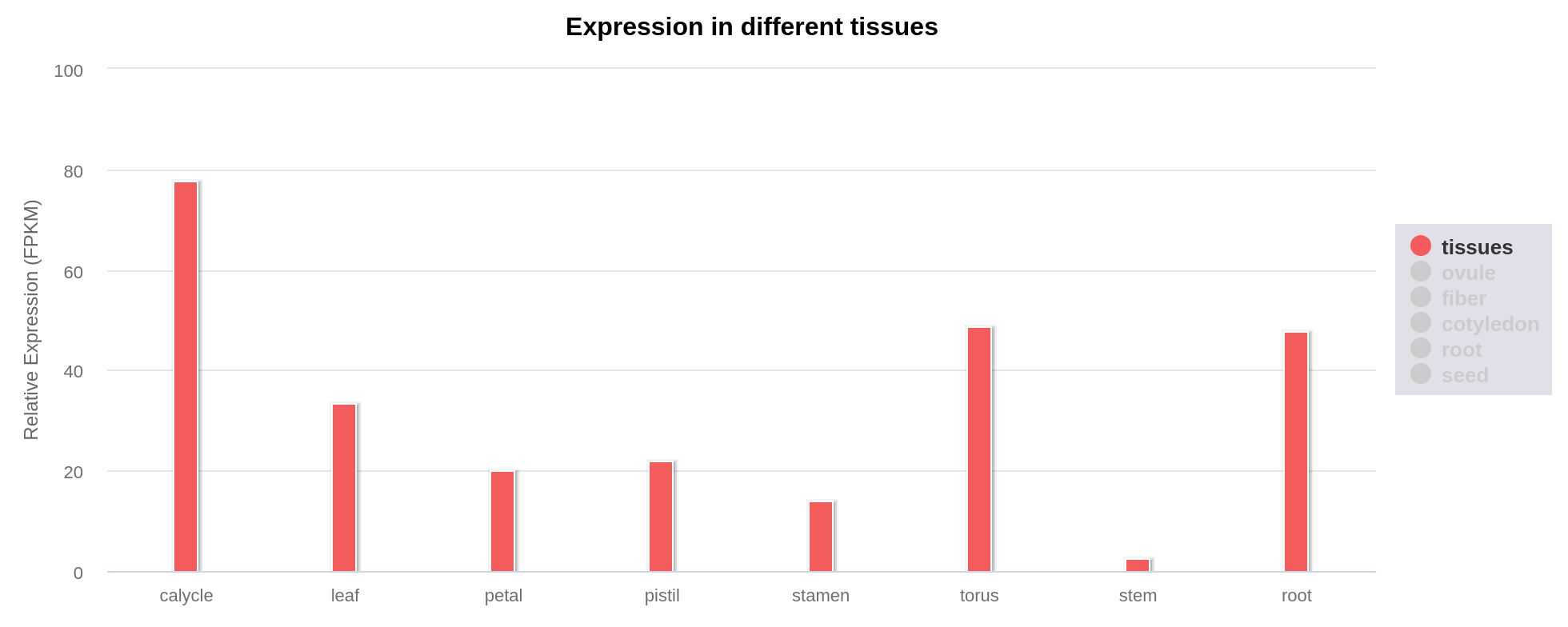

Supplement: Supplementary file 1 [file plants-13-01509-s001.zip › figure.S2 The Expression Pattern of GH_D03G0586 in Cotton Omics Database.jpeg]

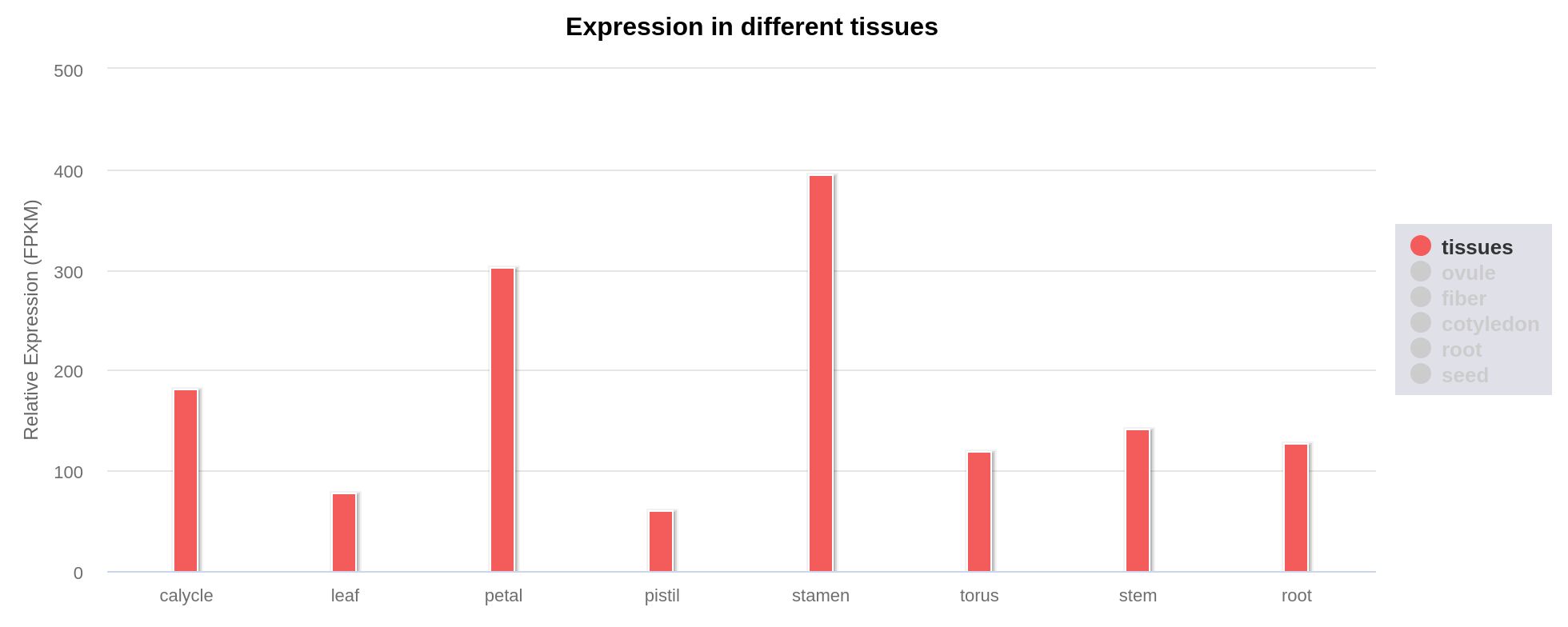

Supplement: Supplementary file 1 [file plants-13-01509-s001.zip › figure.S3 The Expression Pattern of GH_A01G1023 in Cotton Omics Database.jpeg]

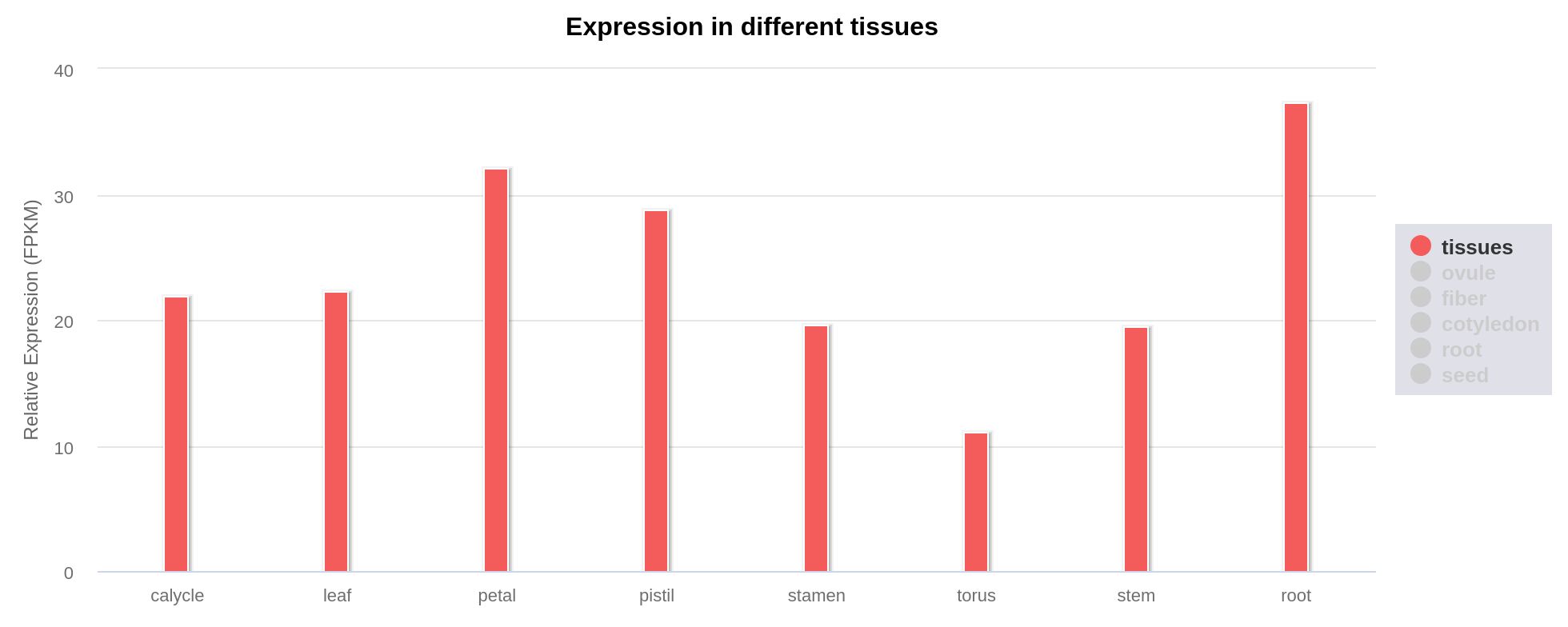

Supplement: Supplementary file 1 [file plants-13-01509-s001.zip › figure.S4 The Expression Pattern of GH_A01G1055 in Cotton Omics Database.jpeg]

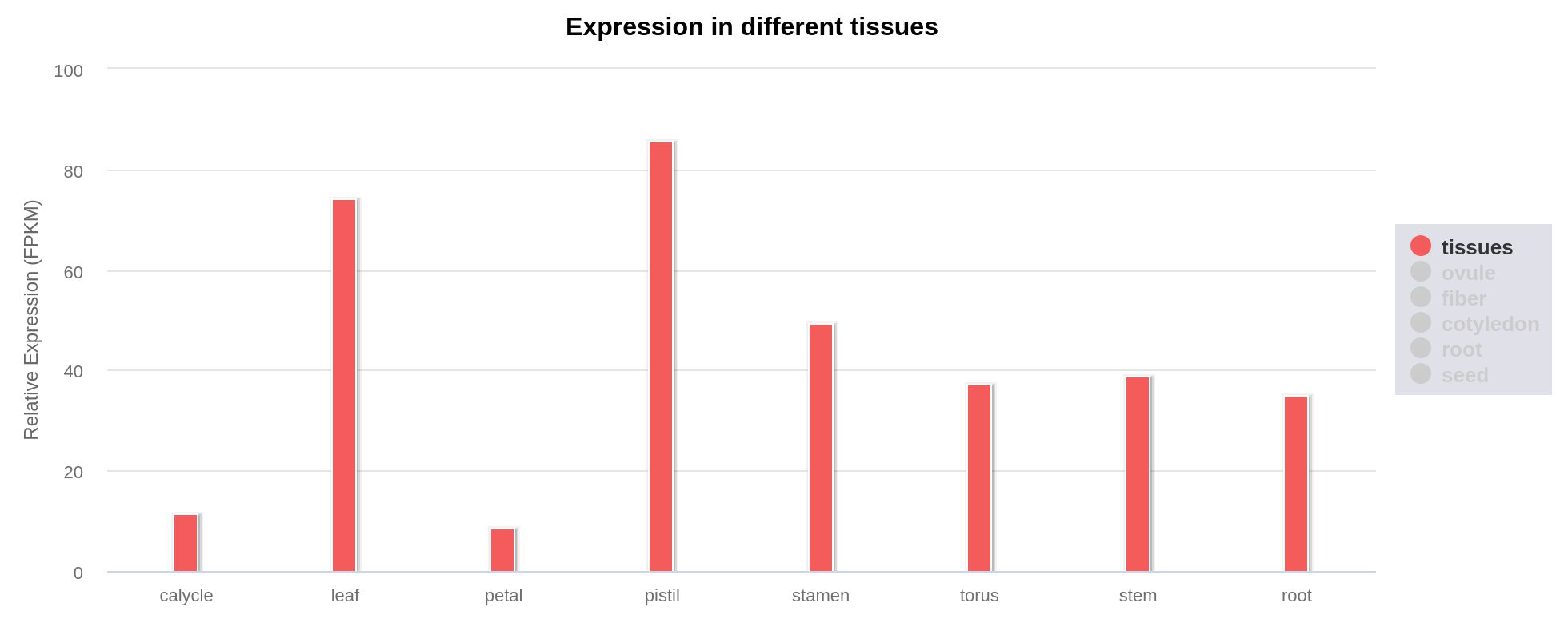

Supplement: Supplementary file 1 [file plants-13-01509-s001.zip › figure.S5 The Expression Pattern of GH_D03G1142 in Cotton Omics Database.jpeg]

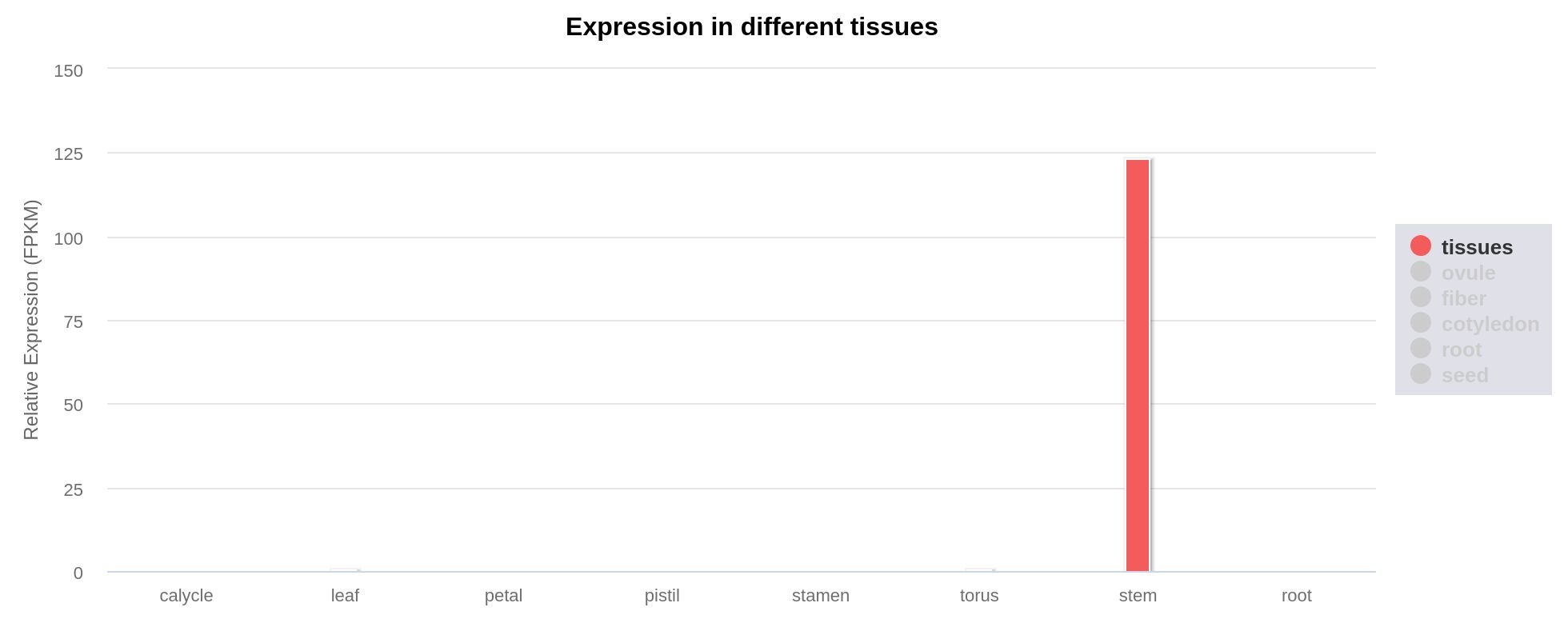

Supplement: Supplementary file 1 [file plants-13-01509-s001.zip › figure.S6 The Expression Pattern of GH_A13G0765 in Cotton Omics Database.jpeg]

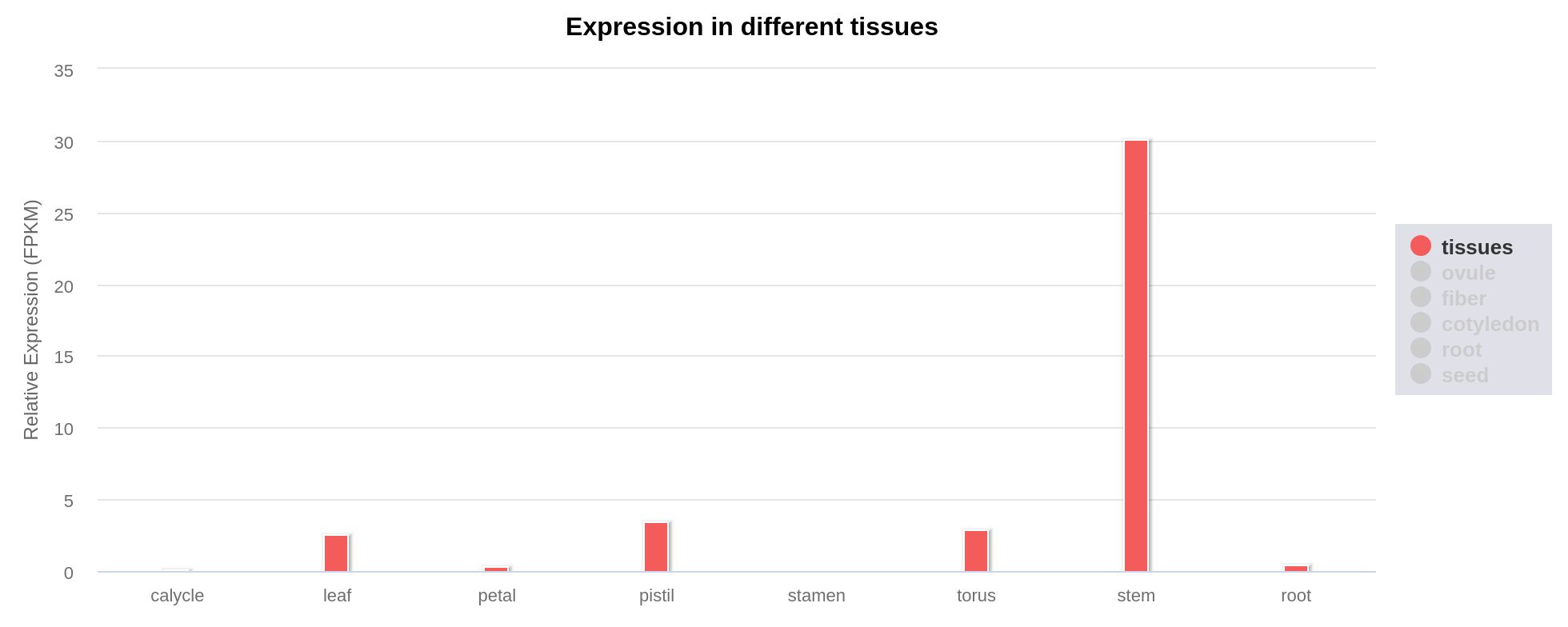

Supplement: Supplementary file 1 [file plants-13-01509-s001.zip › figure.S7 The Expression Pattern of GH_D07G0421 in Cotton Omics Database.jpeg]
